# Effect of VEGF Stimulation on CD11b Receptor on Peripheral Eosinophils in Asthmatics

**DOI:** 10.3390/ijms24108880

**Published:** 2023-05-17

**Authors:** Krzysztof Gomułka, Maciej Tota, Kacper Brzdąk

**Affiliations:** 1 Clinical Department of Internal Medicine, Pneumology and Allergology, Wroclaw Medical University, ul. M. Curie-Skłodowskiej 66, 50-369 Wrocław, Poland; 2 Student Scientific Group of Adult Allergology, Wroclaw Medical University, ul. M. Curie-Skłodowskiej 66, 50-369 Wrocław, Poland

**Keywords:** asthma, CD11b, eosinophils, vascular endothelial growth factor

## Abstract

Asthma is a chronic, complex disease associated with heterogeneity in molecular pathways. Airway inflammation with different cell activation (e.g., eosinophils) and with hypersecretion of many cytokines (e.g., vascular endothelial growth factor—VEGF) might be relevant for asthma pathogenesis and responsible for airway hyperresponsiveness and remodeling. The aim of our study was to reveal the expression of activation marker CD11b on peripheral eosinophils unstimulated and after VEGF in vitro stimulation in asthmatics with different degrees of airway narrowing. The study population included a total of 118 adult subjects: 78 patients with asthma (among them 39 patients with irreversible bronchoconstriction and 39 patients with reversible bronchoconstriction according to the bronchodilation test) and 40 healthy participants as a control group. CD11b expression on peripheral blood eosinophils was detected in vitro using the flow cytometric method without exogenous stimulation (negative control), after N-formyl-methionine-leucyl-phenylalanine stimulation (fMLP; positive control) and after stimulation with VEGF in two concentrations (250 ng/mL and 500 ng/mL). CD11b marker was slightly presented on unstimulated eosinophils in asthmatics and the subgroup with irreversible airway narrowing (*p* = 0.06 and *p* = 0.07, respectively). Stimulation with VEGF enhanced the activity of peripheral eosinophils and induced CD11b expression in asthmatics in comparison with a healthy control (*p* < 0.05), but it was dependent neither on the concentration of VEGF nor on the degree of airways narrowing in patients with asthma. We present our findings to draw attention to the potential role of VEGF in the eosinophil priming and CD11b-mediated signaling in patients with asthma which is currently undervalued.

## 1. Introduction

Asthma is a chronic airway disorder affecting many people globally and is considered a complex disease in which hypersecretion of many cytokines (e.g., growth factors) and various cells activation (e.g., eosinophils) determines its different endotypes. This heterogeneity in molecular pathways is related to smooth muscle hypertrophy, subepithelial fibrosis, vascular leak, and angiogenesis, contributing to bronchial hyperresponsiveness and a variable degree of airflow obstruction [1,2,3].

Vascular endothelial growth factor (VEGF) is a homodimer, heparin-binding glycoprotein with an essential pro-angiogenic activity that elevates vascular permeability, stimulates cell migration, and retains mitogenic and angiogenic effects on endothelial cells. The VEGF family comprises the following members: VEGF-A (simply called VEGF), VEGF-B, VEGF-C, VEGF-D, VEGF-E (viral VEGF), VEGF-F (snake venom VEGF), PIGF (placenta growth factor), and EG-VEGF (endocrine gland-derived VEGF). VEGF is synthesized mainly by epithelial cells, platelets, neutrophils, and macrophages, and then triggers cell responses by binding to the tyrosine kinase receptors (VEGFR). Whereas vascular endothelial cells mainly express VEGFR-1 and VEGFR-2, lymphatic endothelial cells predominantly present VEGFR-3 [4,5]. Recent studies have reported that VEGF is a key regulator of vascular development and vessel function not only in health (i.e., wound healing, menstrual cycle, endothelial cells growth, reproductive functions, embryogenesis), but also in various diseases (i.e., age-related macular degeneration, diabetic retinopathy, chronic heart disease, rheumatoid diseases, neoplasm, chronic obstructive lung disease) [6,7]. Furthermore, among patients with asthma, VEGF is considered one of the important agents that cause vascular leakage and permeability, as well as endothelial cell proliferation and differentiation. It has been linked to accelerated allergy sensitization, elevated following type 2 inflammatory responses, chemotaxis of monocytes and eosinophils with lung remodeling [8].

Eosinophils account for approximately 0.5–1% of the peripheral leukocytes, and the detection of these activated cells by indication of different protein markers on the membrane (e.g., CD11b, CD69) using the flow cytometry has been shown to be a useful diagnostic tool [9,10]. The eosinophilic phenotype is exhibited in approximately 50% of people with severe asthma [11,12]. It is driven by type 2 inflammation that includes both the innate (type 2 innate lymphoid cells—ILC2) and adaptive immune system (type 2 T-helper—Th2 cells). ICL2 and Th2 cells secrete interleukin (IL)-4, IL-5, and IL-13, which are of pivotal significance in the pathogenesis of asthma. IL-4 and IL-13 induce B-cell class switching, IgE production, pro-inflammatory markers secretion, and tissue remodeling. IL-13 is affected in mucus production, as well as in smooth muscle and goblet-cell hyperplasia [13]. IL-5 mediates eosinophils’ maturation, proliferation, activation, and migration [14]. Moreover, there is strong evidence that VEGFR is expressed on the membrane, and eosinophil activation by VEGF stimulates directed chemotaxis, migration, and the release of the eosinophil cationic protein [15].

**Figure 1:**
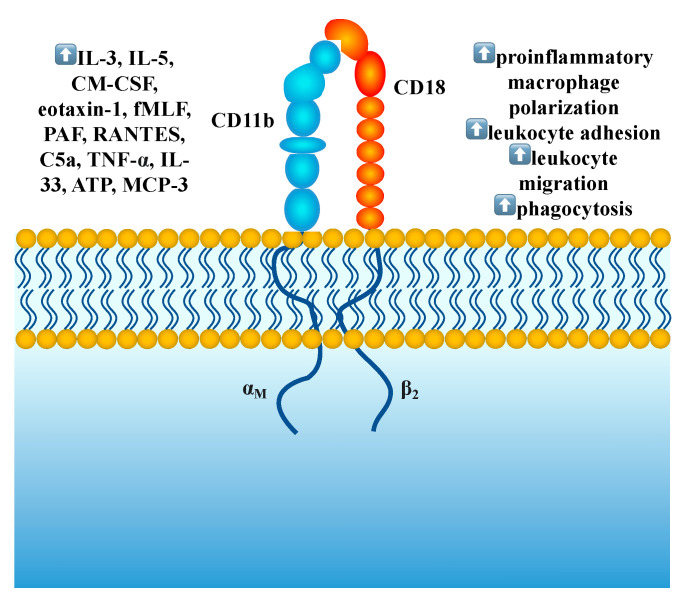
Molecules that upregulate (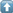) Cd11b/CD18 expression are presented on the left; biological functions of CD11b/CD18 are shown on the right.

CD11b (cluster of differentiation 11b) encoded by the ITGAM gene is also known as integrin subunit α-M, CR-3α (complement receptor 3α), α subunit of MAC-1 (Macrophage-1 antigen). CD11b, in combination with CD18, forms an αMβ2 heterodimer which has been found on the surface of eosinophils, monocytes, macrophages, dendritic cells, neutrophils, natural killer cells, and a subset of B and T cells [16,17,18]. Complex CD11b/CD18 regulates cell adhesion, migration, phagocytosis, and pro-inflammatory cell polarization being over-expressed in various diseases, such as chronic tuberculosis, rheumatoid arthritis, urticaria, atopic dermatitis, eosinophilic esophagitis, sepsis, or Kawasaki vasculopathy [19,20,21,22,23,24,25] (Figure 1).

In the present study, we investigated the expression of the CD11b activation marker on peripheral eosinophils unstimulated and after VEGF in vitro stimulation in asthmatics with different degrees of airway narrowing (reversible and irreversible bronchoconstriction). Despite the growing understanding of the pathomechanism of asthma, to the best of our knowledge, there are no data about this phenomenon in the current literature.

## 2. Results

**Figure 2:**
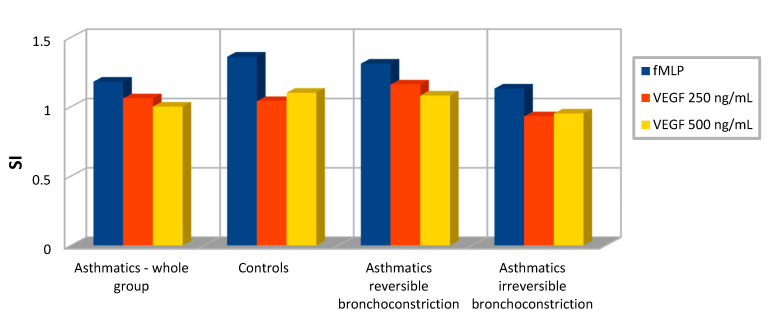
CD11b expression on eosinophils in examined groups—values shown as a Stimulation Index (SI).

To determine the expression of CD11b in examined asthmatics and controls, eosinophils from peripheral blood were incubated with a medium (as a negative control), *N*-formylmethionyl-leucyl-phenylalanine—fMLP (as a positive control) or a different concentration of vascular endothelial growth factor—VEGF (data are shown in Table 1 and on Figure 2).

### 2.1. Effect of the Medium on CD11b Expression

Freshly isolated eosinophils expressed CD11b and culture in the medium had a minimal effect on this marker, and the specific fluorescence of active CD11b^+^ eosinophils was at a similar level in all tested participants. It is worth noting that CD11b marker was slightly presented on unstimulated eosinophils in asthmatics and the subgroup with irreversible airway narrowing in comparison to the control (*p* = 0.06 and *p* = 0.07, respectively).

### 2.2. Effect of fMLP on CD11b Expression

After stimulation of fMLP, expression of the CD11b marker was higher than in a non-stimulated negative sample for all examined groups. However, the specific fluorescence of active CD11b^+^ eosinophils was at a similar level in all tested participants. This difference was not statistically significant (*p* > 0.05).

### 2.3. Effect of VEGF on CD11b Expression

To investigate whether CD11b overexpression could be induced by VEGF, eosinophils were incubated with this cytokine in concentrations of 250 ng/mL and 500 ng/mL for 30 min. There was no significant dose-dependent induction of CD11b expression on eosinophils by VEGF. A statistically significant difference (*p* < 0.05) in a median of activated CD11b^+^ eosinophils was observed only between asthmatics and controls for VEGF stimulation in a concentration of 250 ng/mL. Additionally, obtained results did not show differences (*p* > 0.05) in a level of active CD11b^+^ eosinophils, independently from reversible or irreversible airway narrowing.

## 3. Discussion

An inherent feature of asthma is a chronic inflammatory process in the airways in which activated cells, mainly eosinophils, neutrophils, mast cells, and T helper lymphocytes (Th2 and Th17), participate. Each of these cells might release some cytokines, interleukins, or growth factors—over one hundred various mediators have been recognized so far to be involved during asthma, contributing to symptoms and progressive remodeling processes in the airways [26,27]. Detection of the degree of cell activation and additional plasma markers related to the inflammatory process of the airways in asthmatics in vitro or in vivo tests will make it possible to determine the phenotype and endotype of the disease, the intensity of the remodeling process, as well as to optimize and personalize the patient’s treatment. Additionally, biomarkers taken from peripheral blood from asthmatics are easy and harmless to obtain but this procedure might be more invasive than induced sputum eosinophilia or bronchoalveolar lavage (BAL). What is more, the dynamic recruitment of activated immune cells from the peripheral blood might be assessed as an indirect readout of the disease state [28].

In the available literature, some studies have shown that cells, including granulocyte subpopulations, respond to inflammatory signals by upregulation and overexpression of membrane activation markers in the course of chronic diseases, e.g., asthma. Several of these membrane markers (among others, CD11b, CD203c, and CD69) might be found in cells’ granules shortly after activation by inflammatory mediators and then fused with the membrane and detected several hours after stimulation [29,30,31]. Furthermore, the same phenotype of surface markers was revealed on blood cells and tissue cells from sputum and bronchoalveolar lavage, indicating cells homing to the lung [32]. Interestingly, previously it has been shown that increased VEGF serum concentration applies to patients with asthma, especially with irreversible bronchoconstriction [33]. Other papers suggested basophils or neutrophils VEGF-activation in asthmatics [34,35].

In our current study, we revealed that peripheral blood eosinophils from patients with asthma demonstrated initially, without stimulation, a slightly higher level of activation expressed as the presence of a CD11b marker on their surface. This early activation, called “priming” or “stand-by”, connected with increased cell metabolic activity, might be associated with a greater predisposition to release mediators by various factors—both inner (e.g., cytokines) and outer (e.g., allergens). Moreover, we also observed CD11b overexpression on fMLP-stimulated eosinophils in asthmatics. It might indicate the eosinophil’s activity affection by various unspecific agents—in the ongoing inflammatory process in asthmatics, exposure to environmental pollution, cigarette smoke, or respiratory tract infections should also be considered [2]. Additionally, if eosinophils present higher susceptibility to synthesis and degranulation of pro-inflammatory factors, priming may be associated with symptom severity and efficient treatment of asthma. Increased numbers of blood and tissue eosinophils have been observed in subjects suffering from asthma and play a role in airway inflammation and exacerbation of this disease. Peripheral blood eosinophils being in different activation states, including pre-activated or primed, may circulate to the airway endothelium and be closely associated with clinical symptoms then leading to airway narrowing, concomitant tissue damage by released proteins, or remodeling in asthmatics [10,36,37].

Furthermore, obtained data indicate that stimulation of blood eosinophils by VEGF leads to higher CD11b expression and enhances the activity of these cells (mostly in patients with asthma and reversible airway narrowing) in comparison with the unstimulated sample. A significant difference in the degree of activation of eosinophils after stimulation by VEGF (in a concentration of 250 ng/mL) was found between the entire study group of asthmatics and the control group. However, different degrees of airway narrowing or increasing VEGF concentrations did not lead to significantly higher CD11b expression on the eosinophils membrane in examined groups of patients. In this context, it can be assumed that VEGF leads to upregulation of CD11b expression and increases in eosinophils activity, hardly anyone has described this phenomenon in the available literature [38,39]. On the other hand, it should be noted that VEGF is unlikely to activate eosinophils in a dose-dependent manner and is rather not a leading factor evoking eosinophils activation in asthmatics. Additionally, CD11b might be a more valuable marker associated with cell adhesion and transmigration or asthma exacerbation—future evaluation in this direction of patients with obstructive airway diseases might be important in everyday clinical practice [5,40].

Some potential limitations of our study may include the use of only one membrane marker to determine the degree of eosinophil activation by VEGF and a comparatively small size of the investigated groups of participants. Therefore, we believe that our work needs further examinations conducted not only in one center but also extending to a phenotyping panel with other eosinophil receptors and other simulating concentrations of VEGF or/and other cytokines on a greater sample of asthmatics with the initial phase, during progression or exacerbation of asthma.

## 4. Materials and Methods

### 4.1. Study Groups

The study population included a total number of 118 individuals (aged from 20 to 70 years; 78 females) who signed informed consent to participate in our study. Among the study group, 78 patients (aged from 23 to 69 years; 52 females) had the diagnosis of asthma established earlier according to criteria defined in The Global Initiative for Asthma (GINA) recommendation [41]. After a standard bronchodilation test with salbutamol, asthmatics were divided into two cohorts: 39 patients with reversible airway obstruction (aged from 23 to 69 years; 28 females) and 39 patients with irreversible airway obstruction (aged from 24 to 69 years; 24 females). Among asthmatics with reversible airway narrowing, 36 participants used inhaled corticosteroids (fluticasone propionate or budesonide), and 37 patients were treated with long-acting beta-2 agonists (formoterol fumarate or salmeterol). In the group of asthmatics with irreversible airway obstruction, 25 participants used inhaled corticosteroids (fluticasone propionate or budesonide), and 26 patients were treated with long-acting beta-2 agonists (formoterol fumarate or salmeterol). None of the asthmatics used systemic corticosteroids during the study. The control group consisted of 40 volunteers (from 20 to 70 years; 26 females) who were healthy and without chronic pulmonary dysfunction or allergies in their medical history. The exclusion criteria for patients with asthma and healthy controls included: lack of consent, age under 18 or over 70 years, asthma and chronic obstructive pulmonary disease overlap (ACO), asthma exacerbation, significant comorbidities (e.g., neoplasm), substance use disorder, pregnant and breastfeeding females. This study was conducted in accordance with the Declaration of Helsinki, and the study protocol was approved by the local ethics committee (protocol code KB—68/2011). The population data of all examined groups are shown in Table 2.

### 4.2. Bronchodilation Test

Pulmonary function and bronchodilation tests were performed in all patients with asthma. Forced vital capacity (FVC) and forced expiratory volume in 1 s (FEV1) values were established using a Master Scope CT Spirometer (Erich Jaeger GmbH, Wurzburg, Germany) and presented as a percentage of predicted values (% predicted). Each parameter was taken 3 times, and the best score was used in further analysis. The bronchodilation test was performed after inhalation of 400 μg of salbutamol (SteriNeb Salamol, Teva Pharmaceuticals, Warsaw, Poland). Reversible bronchoconstriction was defined as increases in postbronchodilator values (FEV1 > 120 mL and >12%).

### 4.3. Flow Cytometry Method

In order to investigate CD11b expression, peripheral eosinophils were cultured with medium (non-stimulated negative control—patient background: Pb), *N*-formylmethionyl-leucyl-phenylalanine (fMLP in a concentration of 10^−6^ M—positive control: Pc), and VEGF (two samples in concentrations of 250 ng/mL and 500 ng/mL). Initially, all samples contained 100 μL blood taken to the 4.5 mL tubes with lithium heparin (Sarstedt AG & Co., Nümbrecht, Germany) and 100 μL RPMI-1640 Medium (Institute of Immunology and Experimental Therapy, Wroclaw, Poland), 100 μL fMLP (Institute of Immunology and Experimental Therapy, Wroclaw, Poland), or 100 μL VEGF (BD Biosciences Pharmingen, San Diego, CA, USA). Samples were incubated for 60 min in the atmosphere supplemented with 5% CO_2_ at 37 °C (Incubator ASSAB, Stockholm, Sweden). In the next step, 20 μL of edentate disodium EDTA (BD Biosciences Pharmingen, San Diego, CA, USA) was added, and samples were centrifuged for 10 min at 1600 rpm Then, the supernatant was removed, 100 μL of PBS (Institute of Immunology and Experimental Therapy, Wroclaw, Poland) with 1% bovine serum albumin (Sigma-Aldrich, St. Louis, MO, USA) and 10 μL of anti-CD11b (Immunotech S.A.S., Marseille, France) were added and incubated in the darkness at 25 °C for 30 min. After the incubation process, 2 μL of fluid for the cell lysis (BD Biosciences Pharmingen, San Diego, CA, USA) was added and after 10 min at room temperature the samples were centrifuged at 1600 rpm for 5 min. Afterwards, the supernatant was removed, 3 mL of PBS was added to each sample and centrifuged for 5 min. at room temperature at 1600 rpm. For the cell’s preservation, 200 μL of PBS with 1.5% paraformaldehyde (Sigma-Aldrich, St. Louis, MO, USA) was added. From each examined sample, cells were collected using a FACScan flow cytometer (Becton Dickinson, San Diego, CA, USA). The mean fluorescence of the eosinophil’s population was calculated, and active cells were identified—the expression of CD11b on the eosinophil surface was used for the analysis of their activity. The obtained results were further analyzed as median (Me) with minimum and maximum values (min.; max.) of samples intensity. Findings were presented as Stimulation Index (SI), which is the quotient of the fluorescence intensity after stimulation to the fluorescence intensity of the primary sample without exogenous stimulation (negative control). Procedures of eosinophil identification described above were coincident with the accepted methods [42,43].

### 4.4. Statistical Analysis

All statistical analyses were conducted by the Statistica Software Package, version 10 (Polish version; StatSoft, Kraków, Poland). Comparisons between examined groups were performed using the Kruskal–Wallis ANOVA test. The level of statistical significance was set at a *p*-value < 0.05.

## 5. Conclusions

We present our findings to draw attention to the potential role of VEGF in eosinophil priming and activation in patients with asthma, which is currently undervalued. In summary, this study found that VEGF might slightly modulate the expression of the CD11b marker on peripheral eosinophils in asthmatics. However, it is related neither to the concentration of VEGF nor the degree of airway narrowing. These outcomes are consistent with the results of other studies, which imply that various immune cell types and cytokines might be important for asthma endotypes definition. CD11b-mediated signaling might be the goal of future pharmacotherapy in asthma as an inflammatory disease. However, these results should be further addressed in a larger study group.

## Figures and Tables

**Table 1:** CD11b expression on eosinophils from examined groups—values shown as a specific fluorescence—presented as the median value (minimum; maximum).

Incubation Placement	Asthmatics			Controls
	All Examined Patients	Group with Reversible Airway Narrowing	Group with Irreversible Airway Narrowing	
Patient background (Pb—medium)	332.21 (135.67; 725.60)	300.27 (151.24; 676.32)	347.56 (135.67; 725.60)	297.76 (8.42; 886.62)
Positive control (Pc—fMLP)	393.10 (206.18; 1619.68)	394.15 (206.18; 905.30)	392.05 (218.74; 1619.68)	405.98 (17.74; 2181.30)
VEGF (250 ng/mL)	350.86 * (127.59; 1003.32)	335.10 (127.59; 668.25)	322.68 (144.21; 1003.32)	309.48 * (5.66; 1158.14)
VEGF (500 ng/mL)	331.53 (157.80; 1077.13)	323.02 (163.03; 764.08)	329.51 (157.80; 1077.13)	327.22 (9.13; 1507.55)

* *p* < 0.05 (by Kruskal–Wallis ANOVA test); fMLP—*N*-formylmethionyl-leucyl-phenylalanine; VEGF—vascular endothelial growth factor.

**Table 2:** The demographic data and clinical profiles of examined groups.

Variables	Asthmatics		Controls
	Reversible Airway Narrowing	Irreversible Airway Narrowing	
Participants (N)	39	39	40
Female gender (%N)	28 (71.79%)	24 (61.54%)	26 (65%)
Age (years) mean ± SD	50.0 ± 12.7	51.7 ± 10.3	48.0 ± 13.7
Age (years) Me (min.; max.)	51.0 (23; 69)	54.0 (24; 69)	52.5 (20; 70)
Asthma duration (years) mean ± SD	10.04 ± 8.28	21.94 ± 11.88	-
inhGKS (%N)	36 (92.31%)	25 (64.10%)	-
LABA (%N)	37 (94.92%)	26 (66.67%)	-
Smoker—current or former (%N)	5 (12.82%)	14 (35.9%)	11 (27.50%)

N—number of participants; SD—standard deviation; Me (min.; max.)—median (minimum; maximum); inhGKS—inhaled corticosteroids; LABA—long-acting beta-2 agonists.

## Data Availability

The data that support the findings of this study are available from the corresponding author upon request.
